# Scale-Agnostic Models Based on Dimensionless Quality by Design as Pharmaceutical Development Accelerator

**DOI:** 10.3390/ph18071033

**Published:** 2025-07-11

**Authors:** Miquel Romero-Obon, Virginia Sancho-Ochoa, Khadija Rouaz-El-Hajoui, Pilar Pérez-Lozano, Marc Suñé-Pou, Josep María Suñé-Negre, Encarna García-Montoya

**Affiliations:** 1 Department of Pharmacy and Pharmaceutical Technology and Physical Chemistry, Faculty of Pharmacy and Food Sciences, University of Barcelona, Av. Joan XXIII, 27-31, 08028 Barcelona, Spainkhadijarouaz@ub.edu (K.R.-E.-H.); perezlo@ub.edu (P.P.-L.); marcsune@ub.edu (M.S.-P.); jmsune@ub.edu (J.M.S.-N.); 2 Pharmacotherapy, Pharmacogenetics and Pharmaceutical Technology Research Group, Bellvitge Biomedical Research Institute (IDIBELL), Av. Gran via de l’Hospitalet, 199-203, 08090 Barcelona, Spain

**Keywords:** Quality by Design, design space, Pi–Buckingham, dimensional analysis, design of experiments, pharmaceutical process development

## Abstract

This comprehensive review of the synergistic use of Quality by Design (QbD) and the Pi–Buckingham theorem explores an innovative approach to enhancing product development and process optimization within the pharmaceutical industry. QbD is a systematic, proactive methodology that integrates quality considerations throughout the product lifecycle to ensure that pharmaceutical products meet regulatory standards for safety and efficacy from the outset of development. The Pi–Buckingham theorem serves as a foundational principle in dimensional analysis, facilitating the simplification of complex models by transforming physical variables into dimensionless parameters. This synergy enables researchers to better understand and control the factors affecting critical quality attributes (CQAs), thereby improving manufacturing outcomes and minimizing variability.

## 1. Advantages and Opportunities of Combining Quality by Design with the Pi–Buckingham Theorem

The integration of QbD and the Pi–Buckingham theorem can address significant challenges in pharmaceutical development, such as the need for efficient experimental designs and comprehensive risk assessments. By employing the principles of the Pi theorem, practitioners can systematically identify the relationships between process variables and quality attributes without extensive empirical testing, leading to more streamlined processes and robust product development strategies. This collaborative approach not only fosters communication among multidisciplinary teams but also aligns with regulatory expectations, promoting a culture of quality and continuous improvement within the industry [1,2,3].

The Pi–Buckingham theorem is a systematic method for reducing the number of variables in a physical problem by identifying dimensionless groups, thereby facilitating the analysis of complex systems via dimensional analysis. When integrated with the foundational principles of QbD, this theorem offers significant advantages through parameter reduction, such as minimizing the number of experiments required to achieve an equivalent level of process understanding and enabling the decoupling of the design space from a fixed batch size.

The mathematical models that constitute the dimensionless Design Space are independent of scale, facilitating applicability from laboratory and/or pilot scale to commercial scale, provided that geometric similarity is maintained and there are no fundamental technological changes. This fact is crucial for accelerating the development and scalability of the process.

Despite these advantages, the implementation of this methodology presents notable challenges. A critical hurdle is achieving a deep understanding of both product and process dynamics, which is essential for effective QbD applications. Moreover, the complexity and subjectivity of the methodologies employed in risk assessment and design of experiments can act as barriers to successful implementation, necessitating a solid foundation in statistical theory and extensive knowledge of pharmaceutical development for accurate interpretations. As the pharmaceutical landscape evolves, commitment to adaptability and continuous improvement remains imperative to navigate regulatory changes and advancements in scientific knowledge.

In summary, the synergistic use of QbD and the Buckingham Pi theorem represents a transformative approach in the pharmaceutical industry, enhancing the development and optimization of products while ensuring compliance with stringent quality requirements. The exploration of this integration highlights the potential for innovative practices that can redefine traditional methodologies and promote greater efficiency in product development [1,4,5].

## 2. State of the Art in the Pharmaceutical Industry and Other Sectors

QbD is a fundamental concept in the pharmaceutical industry that emphasizes a proactive approach to quality assurance, ensuring that products are designed with quality in mind from the outset [6,7,8]. This paradigm shift, first articulated by quality expert Joseph M. Juran in 1992, aims to embed quality into the development of processes to consistently deliver safe and effective products that meet regulatory standards [9].

A key aspect of QbD is understanding the various factors that can impact the quality of pharmaceutical products. These factors include the composition of the input materials, process parameters, environmental conditions like temperature, and human factors such as operator skill. Ensuring consistency in these areas is essential for maintaining control over the manufacturing processes and minimizing defects.

**Figure 1:**
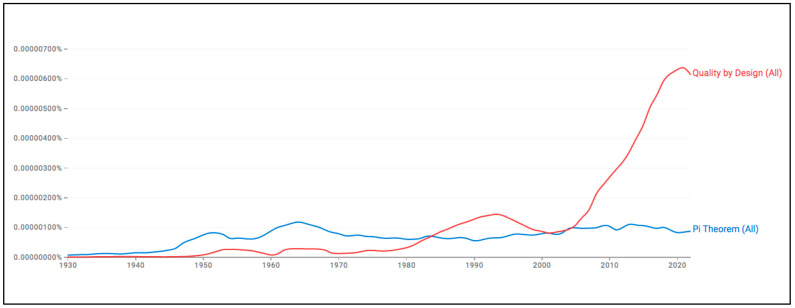
Relative prevalence of the use of the “Quality by Design” and “Pi Theorem” terms in publications from 1930 to 2022. While the Pi Theorem remains stable over the years, QbD shows a peak matching Juran’s book “Quality by Design” (1992) and a significant increase after the first release of ICH Q8 (2005). The graphic was created by the authors using Google N-grams (http://books.google.com/ngrams/, Data set updated July 2024, query performed 13 April 2025).

QbD was swiftly adopted across various industrial sectors, with the notable exception of the pharmaceutical industry due to its stringent regulatory environment. Similar to other emerging technologies and concepts that have exhibited temporal lag, QbD has been cautiously implemented only following the publication of the guideline “Q8—Pharmaceutical Development” by the International Council for Harmonization (ICH) in 2005 [6] (see Figure 1).

As the pharmaceutical industry increasingly adopts QbD principles, the importance of integrated methodologies that encompass both statistical analysis and quality design principles is increasingly recognized, highlighting the need for a comprehensive understanding of these concepts for effective implementation in product development.

The pharmaceutical industry ranks lower in priority when it comes to the Pi–Buckingham theorem application. Only a limited number of scientific articles document the combined use of this theorem with Quality by Design (QbD) principles.

In this study, more than 60 bibliographic sources were reviewed and thematically categorized as follows: most of these were published between 2005 and 2025 and included regulatory guidelines from drug agencies, scientific review articles, and original research papers [1,2,3,4,5,6,7,8,9,10,11,12,13,14,15,16,17,18,19,20,21,22,23,24,25,26,27,28,29,30,31,32,33,34,35,36,37,38,39,40,41,42,43,44,45,46,47,48,49,50,51,52,53,54,55,56,57,58,59,60,61,62,63]. The sources were retrieved from the scientific databases Web of Science and PubMed, as well as through the AI-assisted search tool Consensus, using the keywords “QbD + Pi–Buckingham” and “dimensionless QbD”. The thematic classification highlights the diversity of applications of both the Quality by Design (QbD) paradigm and Pi–Buckingham theorem across the pharmaceutical and engineering sectors.

Foundational and developmental works on the Pi–Buckingham theorem, along with dimensional analysis, are represented by both historical and contemporary studies that established the basis for physical modeling and process scale-up methodologies [1,2,4,5]. These foundations have expanded into hybrid approaches that incorporate machine learning and symbolic modeling [16,24,35,40,53,56,61,62,63].

In parallel, the principles of QbD have evolved from key regulatory frameworks and conceptual articles [3,6,7,8,9] into broader implementation in product development, quality control, and pharmaceutical manufacturing [10,11,12,13,14,15], including their integration into the ICH guidelines and regulatory best practices [17,18,19,20,21,22,23,25,26,33].

Notably, the group of references related to pharmaceutical development has been further differentiated based on the prevailing methodological paradigm: on one hand, studies that follow systematic, regulation-driven QbD approaches (ICH Q8–Q14) and, on the other, those that apply dimensional analysis and the Pi–Buckingham theorem as modeling tools in pharmaceutical and biopharmaceutical sciences. This distinction underscores not only the complementarity of both paradigms but also the independent scientific trajectories each has followed in their respective evolution.

Other groups of studies emphasize emerging and innovative applications, including nanomedicine, education, biosimilars, and clinical research, reflecting a broader and forward-looking perspective of QbD [27,29,30,31,32,45,46]. The consolidation of Analytical QbD (AQbD) has also been well documented in several reviews and applied research articles focused on the development of analytical methods [19,20,21,28,34,38,39,54,55].

References addressing quality control strategies, regulatory compliance, and product lifecycle management highlight the value of QbD as a systematic tool for ensuring quality from design to post-commercialization [30,33,36,37,38,44,46,51,55,59,60].

Finally, the most recent publications suggest a convergence between the systematic framework of QbD and advanced modeling approaches, including physical modeling and artificial intelligence, which are applicable even to offshore and structural engineering contexts [11,14,24,32,35,40,41,53,61,62,63]. This convergence points to the emergence of a scale-agnostic methodological framework that is applicable across diverse sectors.

This thematic synthesis is complemented by Supplementary Table S1, which provides detailed information on each reference, including the document type, application area, and employed techniques.

**Figure 2:**
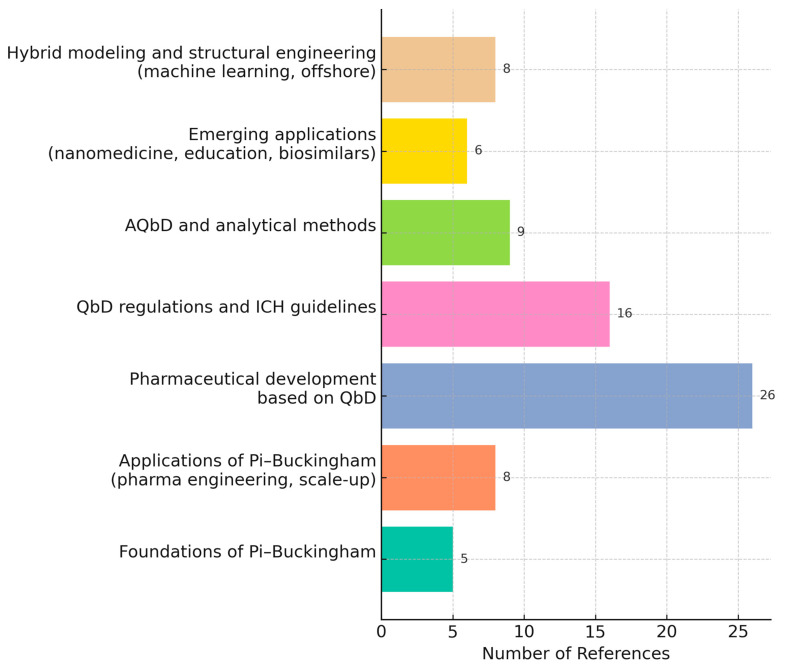
Publications grouped by thematic area and methodological approach (Quality by Design vs. Pi–Buckingham theorem applications). Each bar includes reference numbers [1,2,3,4,5,6,7,8,9,10,11,12,13,14,15,16,17,18,19,20,21,22,23,24,25,26,27,28,29,30,31,32,33,34,35,36,37,38,39,40,41,42,43,44,45,46,47,48,49,50,51,52,53,54,55,56,57,58,59,60,61,62,63] (see Supplementary Table S1 for full details).

An analysis of the number of publications from January 2005 to March 2025 reveals that the Pi–Buckingham theorem and/or QbD principles have been consistently utilized across various sectors (see Figure 2), with significant growth since 2019.

**Figure 3:**
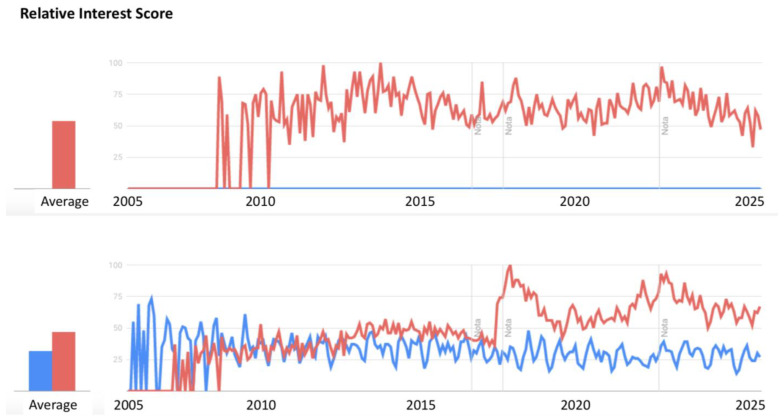
International publications on QbD (red) and the Pi–Buckingham theorem (blue) in the period 2005–2025 in all sectors (**above**) and in the pharmaceutical industry (**below**). Image built by the authors using Google Trends. https://trends.Google.es. Query performed 13 April 2025.

In contrast, within the pharmaceutical industry, the Pi–Buckingham theorem is notably absent (see the bottom graph in Figure 3, where the blue color representing the Pi theorem is zero), whereas QbD principles first appeared in 2008 and have maintained a stable trend up to the present day.

## 3. Traditional QbD Framework

QbD is a systematic and proactive approach aimed at ensuring product quality through a comprehensive understanding of the processes and products involved in pharmaceutical development. This methodology encourages manufacturers to integrate quality considerations from the outset of product development rather than relying on end-of-process testing, thereby addressing the limitations of traditional quality assurance practices (Quality by Testing, QbT) [6,7].

The implementation of QbD has numerous benefits for the pharmaceutical industry. By fostering a deeper understanding of product and process interactions, manufacturers can achieve more consistent product performance, reduce variability, and enhance overall quality assurance practices. Furthermore, QbD aligns with the regulatory expectations set forth by agencies such as the EMA and FDA, promoting a more efficient and reliable pharmaceutical development process [8,51].

### 3.1. Key Elements of QbD

The essential concepts in QbD are Critical Quality Attributes (CQA), Critical Process Parameters (CPP), and Critical Material Attributes (CMAs). CQAs refer to the physical, chemical, microbiological, or biological properties derived from the requirements that define a Quality Target Product Profile. CQAs must be controlled to ensure the desired product quality [6,7].

In contrast, CPPs and CMAs have a direct impact on CQAs; therefore, they must be closely monitored and controlled throughout the manufacturing process. CPPs and CMAs function as explanatory variables within the system of equations that relate them to CQAs. This set of mathematical equations defines the Design Space (DS). Causal relationships can be inferred when the explanatory power of the derived mathematical models is high. These causal inferences enable effective process and product quality control through the appropriate management of input variables, CPPs, and CMAs.

The establishment of the equations that define the DS is based on a prospectively planned Design of Experiments (DoE). The number of experiments required is closely linked to the number of variables acting as the process inputs. Consequently, the dimensionality reduction enabled by the Buckingham π theorem represents a significant advantage, as it reduces the experimental burden while preserving essential relationships among variables.

### 3.2. Risk Assessment

A foundational step in the QbD framework is conducting a thorough risk assessment. This process identifies and evaluates the potential risks associated with the product and manufacturing processes. Tools such as Failure Mode and Effects Analysis (FMEA) are commonly used to prioritize risks based on their potential impact on product quality. Traditional QbD explores the potential CPPs and CMAs among all factors using risk-based decisions by a collective of field specialists [6,7]. In contrast, the approach presented in this article offers a scenario with reduced subjectivity, in alignment with the principles of Risk Management, as outlined in ICH Q9.

### 3.3. Experimental Design

Once risks have been identified, the next phase involves an experimental design. By utilizing DoE, practitioners can systematically investigate the relationships between various process parameters and the resulting CQAs. In the first step, a screening DoE is set and executed to identify the parameters that are the main contributors to CQAs variability among those selected in the previous risk assessment step. This approach not only identifies critical parameters and discards those without statistical significance, but also aids in the optimization of formulations in the next step.

### 3.4. Design Space Definition and Process Optimization

DoE provides essential support to objectively identify non-linearity, interaction existence, and a more fine-tuned set of equations that lead to the DS.

The DS is defined as a multidimensional combination and interaction of input variables that have been demonstrated to provide assurance of quality. This space allows flexibility in manufacturing while maintaining product quality [6,7]. Understanding and validating the design space is critical because it enables manufacturers to adjust processes within specified limits without compromising product integrity.

In the case of reaching adequate explainability, the obtained mathematical models can be used not only for optimization purposes but also for virtualizing a simplification of the real process, allowing what-if scenario simulations (Process Digital Twin).

### 3.5. Monitoring and Continuous Improvement

Post-implementation, the QbD framework emphasizes the continuous monitoring of CPPs, CMAs, and the associated CQAs. Feedback loops should be established to facilitate the ongoing assessment and improvement of analytical methods and processes, ensuring sustained product quality throughout the product lifecycle.

## 4. Pi–Buckingham Theorem

The Pi–Buckingham theorem is a pivotal principle in engineering, applied mathematics, and physics, particularly in dimensional analysis. This ensures that mathematical expressions maintain dimensional homogeneity, thereby allowing for the simplification of complex problems [61]. The theorem is formally derived from Rayleigh’s method of dimensional analysis and states that if a physically meaningful equation involves a certain number of *n* physical variables, it can be reformulated using a set of *p = n − k* dimensionless parameters, denoted as pi-variables, where *k* represents the number of physical dimensions involved [61,62,63].

The principles articulated in the Pi–Buckingham theorem have roots in early scientific inquiry, with notable figures such as Isaac Newton recognizing the value of dimensional analysis in his works, including “Principia Mathematica”. The theorem, proposed by Edwin Buckingham in the early 20th century, has since become a fundamental aspect of problem-solving methodologies across various scientific disciplines, emphasizing the universal structure it provides for understanding physical relationships and designing experiments.

### 4.1. Applications in Physics and Engineering

In practical applications, the Pi–Buckingham theorem is important in fluid mechanics and other engineering disciplines. It aids engineers in predicting the performance of systems, such as jet engines, by deriving dimensionless parameters that simplify the analysis of the involved physical phenomena. This approach not only reduces the complexity of the equations but also facilitates the identification of similar systems, allowing for the comparison and scaling of experimental results.

Given that sectors other than pharmaceuticals are not as heavily regulated, the application of the Pi–Buckingham theorem has been widely adopted, while its applications in the pharmaceutical industry are scarcely known. Having observed the interesting advantages of its integration with QbD, it is considered a highly promising area for drug development and the acceleration of scaling processes inherent to the need to initiate early studies on a small scale and complete them on an industrial scale, where batches will eventually be produced for commercial purposes.

### 4.2. Conceptual Pi–Buckingham Theorem Framework

The foundational idea behind the theorem is that it provides a systematic method for computing sets of dimensionless parameters from given variables, regardless of the specific form of the equation [62]. For instance, the relationship between physical dimensions can often be illustrated through simple geometric examples, such as calculating the area of a circle, where various representations (radius, diameter, and circumference) can be reduced to a single dimensionless form. This ability to transform complex variable interactions into manageable forms is crucial for scientists and engineers alike, as it underpins the development and testing of theoretical models and experimental designs.

### 4.3. Use Case Examples

#### 4.3.1. Tablet Coating in a Conventional Pan Coating System

An application case of the Buckingham Pi theorem is summarized below to illustrate the previously defined formal framework. Daniel Niblett et al. describe the implementation of this approach based on a mechanistic model that interrelates the process conditions involved in tablet coating using a conventional pan coating system [56]. The identified dimensionless groups include the Dimensionless Spray Flux and the Niblett Number, which enable the differentiation of coating processes based on the thermodynamics and kinetics of droplet drying.

These two dimensionless quantities were used to select the process parameters for a set of coating experiments performed over a wide range of multivariate process parameters. A dimensionless Regime Map was created to visualize the effect of the interacting process parameters on the overall tablet appearance quality and defects, such as picking and logo bridging.

#### 4.3.2. Bioprocess Scaling Up

Rendón-Castrillón et al. developed a mathematical model of bioprocess scaling for a two-stage gold bioleaching process [35]. By combining the design of experiments with the Buckingham Pi theorem, a predictive model was formulated using the Carbon/Nitrogen, Carbon/Potassium, and Trace elements/Carbon ratios as dimensionless factors. It was found that the dimensionless factors Carbon/Nitrogen and Carbon/Potassium were correlated with the leaching potential of the fermented broth at 1060 cm^−1^. Based on these results, a non-linear logarithmic model based on dimensionless parameters was proposed to explain the behavior of the system with a correlation coefficient of R^2^ = 0.99, which allows predicting the scaling of the bioprocess.

## 5. Synergistic Use of QbD and Buckingham Pi Theorem

**Figure 4:**
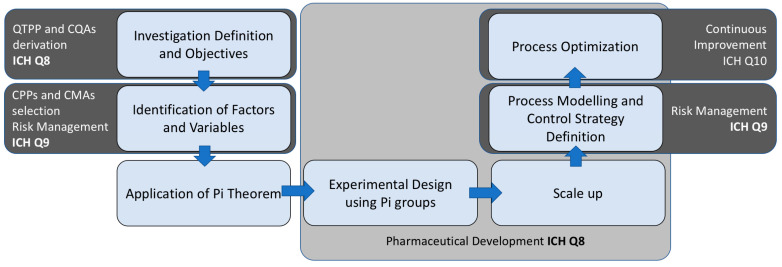
Integration of QbD principles (ICH Q8) with Risk Management (ICH Q9), Continuous Improvement as part of the Quality Management System (ICH Q10), and the Pi–Buckingham theorem.

The integration of the QbD framework with the Buckingham Pi theorem offers a novel approach to enhance product development and process optimization, particularly in the pharmaceutical industry (see Figure 4). The Buckingham Pi theorem provides a systematic method for the nondimensionalization of equations governing physical processes, thereby simplifying complex interactions into dimensionless parameters that can be easily analyzed and compared [62]. This capability allows researchers and engineers to distill the essential factors affecting system behavior, facilitating a better understanding and control of processes.

In the context of QbD, the application of the Buckingham Pi theorem can streamline the identification of CQAs and their relationships with process parameters. QbD emphasizes a proactive approach to quality by systematically identifying and managing the sources of variability that impact quality attributes, thus ensuring product efficacy and safety [6,7].

By utilizing dimensionless groups derived from the Buckingham Pi theorem, practitioners can identify how different process variables interact and influence CQAs without extensive empirical testing. This allows for a more efficient experimental design that can lead to enhanced performance of manufacturing processes, such as the development of modified functioning carriers, where the modeling and application of the theorem can significantly improve outcomes.

The adoption of this methodology offers substantial advantages, most notably a marked reduction in the number of experimental trials required to characterize and optimize the processes. This efficiency gain not only minimizes resource consumption and development time but also enhances the robustness of the process understanding. Furthermore, this approach provides high-resolution thermodynamic insights into the system, enabling a more comprehensive interpretation of the underlying mechanisms. Importantly, this understanding remains valid regardless of the scale of the batch being processed, thereby supporting a scalable and transferable process design.

## 6. Enhancing Product Development Through Synergy

The synergistic use of QbD and the Buckingham Pi theorem not only aids in optimizing existing processes but also paves the way for innovative product development strategies. By applying the principles of nondimensionalization, researchers can explore a wider design space with fewer experiments, ultimately leading to a more robust understanding of product-process interactions. This method aligns with the QbD philosophy of integrating quality into the design phase, thereby minimizing the risk of failure during the later stages of production [6,7].

Moreover, the framework encourages a collaborative approach among multidisciplinary teams, as the dimensionless parameters can serve as a common language to bridge the gaps between engineering, quality assurance, and regulatory affairs.

### 6.1. Benefits

An extensive review of approximately 60 articles identified the following benefits. Notably, most of these beneficial outcomes have been predominantly reported in non-pharmaceutical sectors.

- Reduction of experimental runs during factor screening and process optimization, resulting in time savings and cost efficiency [16,41,56];
- Facilitation of scalability, ensuring a seamless transition from laboratory and pilot to production scale [4,36,40];
- Acceleration of pharmaceutical development, expediting the overall development timeline [24,41];
- Enhancement of technology transfer, promoting efficient and effective transfer of processes and technologies [2,29,63];
- Improved process understanding leads to more robust and reliable manufacturing processes [22,59];
- Enhanced knowledge management fosters better data utilization and decision-making [22];
- Reinforcement of teamwork, encouraging collaborative efforts and interdisciplinary cooperation [2,3];
- Early product release to the market, with advancement of economic benefits and payback to companies and early arrival of new products to clients [16].

### 6.2. Challenges and Considerations

The implementation of this synergistic approach presents several challenges and considerations that practitioners must navigate. A key challenge is achieving a comprehensive understanding of both the product and the process involved in its development, which is crucial for successfully applying QbD methodologies. This necessitates a science-based approach to formulation development and scale-up, integrating various tools and strategies to ensure quality at every stage of the product lifecycle.

One of the fundamental aspects of QbD is the accurate mapping of product attributes to the process parameters. This requires a deep knowledge of the process inputs and their influence on the quality attributes of the product. Establishing a design space through multivariate data analysis and incorporating inline or online Process Analytical Technology (PAT) are essential steps in this process. Furthermore, practitioners must be equipped to utilize statistical methodologies, such as Bayesian statistics, to inform decision-making and enhance process understanding.

In addition to product and process knowledge, effective risk assessment is vital for identifying potential issues that may arise during the development phase. Employing various QbD tools, such as DoE, Risk Assessment frameworks, and mechanistic models, can help predict outcomes and mitigate risks. However, the complexity of these statistical designs can pose a barrier to implementation, requiring a solid foundation in statistical theory and practice to ensure accurate interpretation and conclusions.

One of the challenges associated with the synergistic application of the Buckingham Pi theorem within the QbD framework is the complexity of deriving the original variables into their corresponding dimensionless forms. This task is particularly demanding, given that certain academic disciplines—outside of engineering and physics—do not typically address dimensionless numbers. Therefore, it is essential to establish interdisciplinary teams that effectively integrate specialized knowledge with domain expertise required for pharmaceutical development. Furthermore, the absence of commercial software tools to support this process means that such derivations must often be performed manually by subject-matter experts. An additional challenge is the difficulty in clearly communicating the theoretical foundations and resulting models to regulatory assessors and inspectors, which may hinder the acceptance and implementation of these advanced methods.

### 6.3. Continuous Improvement and Adaptability

As the pharmaceutical landscape evolves, maintaining a commitment to continuous improvement is imperative. This involves regularly revisiting and refining quality practices based on new data and insights from ongoing process analytical studies and capability analyses. The dynamic nature of pharmaceutical development necessitates an adaptable approach to scale-agnostic models, which can accommodate regulatory changes and emerging scientific knowledge by embedding currently well-recognized QbD principles with the dimensionless approach.

## 7. Conclusions

The synergistic use of QbD and the Buckingham Pi theorem represents a transformative approach in the pharmaceutical industry, enhancing the development and optimization of products while ensuring compliance with stringent quality requirements. The exploration of this integration highlights the potential for innovative practices that can redefine traditional methodologies and promote greater efficiency in product development.

Other sectors outside the pharmaceutical industry have initiated modest efforts to combine QbD and the Buckingham Pi theorem. However, no applications have been identified in pharmaceutical process development. The fields where experimentation in this line of work has been predominantly observed include equipment engineering, fluid dynamics, and aeronautics. Significantly less activity is noted in the chemical sector, and finally, the pharmaceutical industry, where the proportion of reviewed scientific articles considering the synergistic approach is less than 2%, according to calculations from the data in Figure 2. Moreover, none of the identified applications have been practically implemented in real pharmaceutical processes for marketed drugs.

## Data Availability

No new data were created or analyzed in this study. Data sharing is not applicable to this article.

## Supplementary Materials

The following supporting information can be downloaded at: https://www.mdpi.com/article/10.3390/ph18071033/s1, Table S1: List of papers published about Pi Buckingham or QbD application ordered by descending year. See the significance of abbreviations at the end of the paper.

## Abbreviations

The following abbreviations are used in this manuscript:

AQbD	Analytical Quality by Design
CMA	Critical Material Attributes
CPP	Critical Process Parameters
CQA	Critical Quality Attributes
DoE	Design of Experiments
DS	Design Space
EMA	European Medicines Agency
FDA	Food and Drug Administration
FMEA	Failure Mode and Effects Analysis
ICH	International Council for Harmonization
PAT	Process Analytical Technology
QbD	Quality by Design
QTPP	Quality Target Product Profile
