# Supplementary material for: Scale-Agnostic Models Based on Dimensionless Quality by Design as Pharmaceutical Development Accelerator
Source: Pharmaceuticals (Basel). 2025 Jul 11;18(7):1033. doi: 10.3390/ph18071033 (PMC12301016; doi:10.3390/ph18071033)
Supplement: Supplementary file 1 [file pharmaceuticals-18-01033-s001.zip › pharmaceuticals-3703197-supplementary.pdf]

Table S1. List of papers published about Pi Buckingham or QbD application ordered by descending year. See the significance of abbreviations at the end of the paper.

Year of Publication	Type of Document	Application	Area / Sector	Technique / Keywords	Reference
2025	Review article	Overview of QbD principles for pharmaceutical product development and regulatory alignment	Pharmaceutical development	QbD, TPP, CQA, design space, control strategy, DoE, PAT	[10]
2024	Research article	Prediction of the dynamic plastic response of metal beams and plates subjected to impact loads	Structural engineering, Materials mechanics	Dimensional analysis, dimensionless number Dg, impact load, transverse deflection, empirical model, experimental validation	[11]
2024	Research article	Discussion on the application of QbD in the pharmaceutical industry to ensure quality in pharmaceutical analysis and product design	Pharmaceutical industry	QbD, QTPP, CQA, CPP, CMA	[12]
2024	Review article	Discussion on the role of QbD in ensuring consistent pharmaceutical product quality through systematic and risk-based development processes	Pharmaceutical development	QbD design, ICH, QTPP, CQA, PAT	[13]
2024	Research article	QbD-based development and optimization of preclinical in vitro assays via experimental design	Preclinical R&D, Assay Development	QbD, design space, experimental design, in vitro assays	[14]
2024	Review article	Comprehensive review of QbD principles, case studies, and future integration with Industry 4.0	Pharmaceutical development	QbD, RTRT, CQAs, CPPs, ICH, Industry 4.0, continuous improvement	[15]
2023	Research article	Simulation of continuous wet granulation using a two-dimensional population balance model predicting both granule size and porosity	Pharmaceutical technology, process engineering	Population balance models (PBM), wet granulation, twin-screw granulation, granule size, porosity	[4]
2023	Research article	Development of an integrated methodology based on dimensional and statistical analysis to build mathematical models from experimental databases	Engineering, applied physics, data science	Dimensional analysis, symbolic regression, genetic programming, neural computing, machine learning, statistical inference	[16]

Year of Publication	Type of Document	Application	Area / Sector	Technique / Keywords	Reference
2023	Review article	Overview of the QbD approach to enhance pharmaceutical product quality and regulatory compliance	Pharmaceutical industry	Quality by Design, ICH Q8/Q9/Q10, USFDA, product quality planning, risk management, pharmaceutical development	[17]
2023	Research article	Overview of QbD concept applied to pharmaceutical product development, formulation, and analytical processes, emphasizing risk management and regulatory guidelines	Pharmaceutical development	QbD, ICH, GMP, TPQP Control strategy, design space	[18]
2023	Review article	Implementation and evaluation of Analytical Quality by Design (AQbD) in pharmaceutical analytical method development	Pharmaceutical analysis and regulation	Analytical Quality by Design (AQbD), Risk assessment, DoE, Method Operable Design Region (MODR), HPLC, UV-Vis, CE, SFC, HPTLC	[19]
2023	Review article	Overview of the increasing relevance of QbD in pharmaceutical product and process development to ensure predefined product quality	Pharmaceutical development	QbD, Holistic approach, Risk-based development, product and process design	[3]
2023	Review article	QbD approach for analytical method development and validation in pharmaceutical quality assurance	Pharmaceutical analysis	QbD, QTPP, QRM, analytical method validation	[20]
2023	Review article	QbD-based formulation and development of nanocarrier drug delivery systems	Nanotechnology / Drug delivery	QbD, DoE, optimization, Box-Behnken, full factorial, ICH Q8–Q14	[21]
2023	Review article	Comprehensive overview of QbD in pharmaceutical and analytical development, including lifecycle management	Pharmaceutical development	QbD, ATPP, DoE, PAT, risk assessment, ICH Q8–Q11, control strategy	[22]
2022	Review article	Overview and practical insights on the application of QbD in pharmaceutical development and manufacturing	Pharmaceutical industry	QbD, ICH guidelines, PAT, product quality profile, traditional vs QbD, process optimization, regulatory compliance	[23]

Year of Publication	Type of Document	Application	Area / Sector	Technique / Keywords	Reference
2022	Research article	Introduction of a machine learning-based framework (dimensionless learning) to derive dominant dimensionless numbers and simplify complex physical systems	Engineering and physical sciences	Dimensionless learning, Machine learning, Dimensional invariance, Scaling laws, Physics-informed modelling, Sparse regression	[24]
2022	Review article	QbD framework for RNA platform process development and regulatory preparedness	RNA therapeutics, Biopharma	QbD, mRNA, CQAs, process modeling, PAT, lipid nanoparticles	[25]
2022	Review article	Academic implementation of QbD in industrial pharmacy research and education	Industrial Pharmacy, Academia	QbD, formulation, quality control, educational methodology	[26]
2022	Review article	QbD implementation in liposomal formulation development for robust and reproducible nanomedicines	Nanomedicine, Drug Delivery	QbD, liposomes, formulation, design of experiments, risk assessment	[27]
2022	Review article	QbD application in the development and optimization of analytical methods	Pharmaceutical analysis	QbD, analytical method development, DoE, risk assessment	[28]
2022	Review article	Application of QbD principles across the pharmaceutical product life cycle	Pharmaceutical development	QbD, control strategy, CMA, CPP, ICH Q8-Q10, design space	[29]
2021	Review article	Practical recommendations for implementing QbD in API process development and scale-up, based on two decades of training and support experience	API process development	QbD, risk analysis, DoE, scale-up, process understanding, control strategy	[30]
2021	Book	Statistical methodologies and study design for biosimilar comparability, switching, and extrapolation	Biopharmaceuticals	Biosimilar development	[31]
2021	Review article	Integration of QbD with QMS to improve design and conduct of clinical studies	Clinical research	QbD, QMS, CTQ, data integrity, clinical study design	[32]
2021	Review article	Regulatory framework and future perspective of QbD with integration of QRM and PAT tools	Pharmaceutical development	QbD, ICH Q8–Q10, PAT, QRM, retrospective QbD, regulatory science	[33]
2021	Book chapter	Risk assessment and design space application in analytical method development using QbD	Pharmaceutical analysis	QbD, risk assessment, design space, ICH Q8–Q11, OOS, OOT	[34]
2021	Research article	Predictive modeling of bioprocess scale-up using DoE and Buckingham Pi theorem in fungal bioleaching	Bioprocess engineering	Bioprocess scale-up, DoE, Buckingham Pi, fermentation, predictive modeling	[35]

Year of Publication	Type of Document	Application	Area / Sector	Technique / Keywords	Reference
2020	Research article	Critical analysis of the current level of implementation of QbD in the pharmaceutical industry and review of key methods and tools	Pharmaceutical industry, Quality management	QbD, risk analysis (FMEA, REM, Ishikawa), design of experiments (DoE), retrospective QbD (rQbD)	[36]
2020	Book chapter	Strategic overview of QbD as a quality tool for pharmaceutical, biotech, and NCE development	Drug development, Biotechnology	QbD, regulatory hurdles, product understanding, process design	[37]
2020	Book chapter	QbD-based development and validation of analytical methods with risk assessment and design space	Pharmaceutical analysis	QbD, analytical method, DoE, risk assessment, method validation	[38]
2020	Conference presentation	QbD-based optimization of liposomal formulations via evaluation of process and formulation variables	Nanomedicine / Drug delivery	QbD, liposomes, vesicle size, DLS, formulation parameters	[39]
2019	Research article	Development and validation of a two-dimensional population balance model to predict liquid content distribution in wet pharmaceutical granules produced using twin-screw granulation	Pharmaceutical technology, process engineering	Two-dimensional PBM, continuous wet granulation, twin-screw granulator, liquid distribution, microcrystalline cellulose, experimental validation	[40]
2019	Conference paper	Establishing the limits of applicability of engineering software using dimensionless numbers for verification and validation	Offshore engineering, energy systems	Dimensionless numbers, similitude laws, verification and validation, engineering judgement, flow assurance, compressibility	[41]
2019	Review article	Implementation of QbD in generic drug development, biologics, and regulatory frameworks	Pharmaceutical and biopharmaceutical industry	Quality by Design, CPP, CQA, validation	[42]
2019	Review article	Overview of QbD as a systematic approach for quality and process understanding	Pharmaceutical development	Quality by Design, Risk management	[43]
2019	Book	Comprehensive implementation of QbD across pharmaceutical development, from formulation to clinical trials	Pharma, Biopharma, Biotechnology	QbD, risk assessment, formulation, stability, bioequivalence	[44]
2019	Review article	Conceptual framework and practical applications of QbD in pharmaceutical product development	Pharmaceutical development	QbD, DoE, PAT, CQA, genotoxic impurities, antidiabetic drugs	[45]

Year of Publication	Type of Document	Application	Area / Sector	Technique / Keywords	Reference
2019	Book chapter	QbD application in the development and manufacturing of biopharmaceuticals and complex formulations	Biopharmaceuticals	QbD, protein production, nanomedicine, formulation development	[46]
2019	Review article	Overview of QbD principles and their role in pharmaceutical product development and manufacturing	Pharmaceutical industry	QbD, ICH Q8-Q10, HACCP, product development, process control	[47]
2019	Review article	General overview of QbD principles and regulatory context in pharmaceutical development	Pharmaceutical development	QbD, ICH guidelines, design space, risk management	[48]
2018	Research article	Development of a methodology to reduce dimensionality in feasibility analysis of the design space for continuous pharmaceutical processes	Pharmaceutical technology, process engineering	Feasibility analysis, design space (DS), PLS regression, dimensionality reduction, continuous direct compaction, RBF, mathematical modelling	[5]
2018	Review article	Overview of the application of QbD in pharmaceutical analysis and manufacturing, including regulatory framework	Pharmaceutical development	QbD, CQA, DoE, risk assessment, ICH Q8-Q10	[49]
2018	Editorial	General overview of the benefits and structure of QbD for pharmaceutical development	Pharmaceutical industry	QbD, ICH guidelines, process control, formulation	[50]
2017	Regulatory report / summary	Evaluation of regulatory experience and convergence in assessing QbD-based submissions in EMA-FDA joint program	Regulatory assessment	Regulatory alignment, QbD review, pilot outcomes, global harmonization	[51]
2017	Research article	QbD application in biopharmaceutical process development using dimensional analysis and machine learning modelling	Biopharmaceutical engineering	Dimensional analysis, machine learning, process modelling, QbD, bioprocess	[1]
2017	Research article	Improvement of enantiomer separation in complex mixtures using ultrafast chiral chromatography in 2D-LC	Analytical chemistry, pharmaceutical analysis	2D-LC, ultrafast chiral chromatography, enantiomer separation	[52]

Year of Publication	Type of Document	Application	Area / Sector	Technique / Keywords	Reference
2017	Research article	Optimal design of experiments for surrogate modelling with dimensionless variables; demonstrated with numerical, thermal, and mechanical cases	Engineering, Numerical optimization	DoE, dimensionless variables, optimization, surrogate models, space-filling design criteria	[53]
2017	Research article	Introduction and implementation of QbD in pharmaceutical manufacturing to improve product quality and process understanding	Pharmaceutical manufacturing	QbD, Risk assessment, DoE, PAT	[54]
2017	Review article	Implementation of QbD in pharmaceutical and biopharmaceutical development and analytical techniques, with emphasis on regulatory context	Pharmaceutical and biopharmaceutical industry	Quality by Design, DoE, Risk assessment, FDA guidance	[55]
2017	Research article	Mechanistic modelling of tablet coating process for prediction of film quality and process optimization	Pharmaceutical manufacturing	Mechanistic modelling, Coating process	[56]
2016	Review article	Comprehensive overview of QbD principles applied to pharmaceutical product development and manufacturing, including regulatory aspects	Pharmaceutical development	QbD, TPP, CQA, design space, PAT, ICH Q8-Q9	[57]
2016	Review article	Introduction to QbD framework for systematic pharmaceutical product and process development	Pharmaceutical development	QbD, QTPP, CQA, CPP, ICH Q8–Q10	[58]
2014	Research article	Application of dimensional analysis in statistical design and analysis to improve understanding of causal relationships and result scalability	Applied statistics, physical sciences and engineering	Dimensional analysis, experimental design, regression analysis, scalability, statistical methodology	[2]
2014	Review article	Clarification and dissemination of the QbD framework in pharmaceutical development	Pharmaceutical development and manufacturing	QTPP, CQAs, CMAs, CPPs, control strategy, DoE, PAT, risk assessment, process design, continual improvement	[59]
2014	Review article	Evaluation of process variables on oral dosage forms using QbD framework	Pharmaceutical manufacturing	QbD, TPP, TPQP, CQA, process parameters	[60]
2012	Regulatory guideline: ICH Q11	Guides the development and manufacture of drug substances under QbD principles (chemical entities)	Drug substance development	Design space, manufacturing process design, control strategy, critical process parameters (CPP), quality risk management	[7]

Year of Publication	Type of Document	Application	Area / Sector	Technique / Keywords	Reference
2009	Regulatory guideline: ICH Q8 (R2)	Provides a harmonized framework for applying QbD principles in pharmaceutical development	Drug development	QbD, design space, critical quality attributes (CQA), risk management	[6]
2009	Web resource	Outlines the EU approach to quality by design and development under EMA's regulatory framework	EU pharmaceutical regulation	QbD, development tools, innovation support, regulatory guidance	[8]
1992	Book	Provides a practical plan using the Quality Planning Roadmap to achieve strategic, market-driven goals through case studies in service, manufacturing, and support sectors	Quality management in service, manufacturing, and support functions	QbD, quality planning map, customer needs, process features, strategic quality goals	[9]
1914	Research article	Formalized the Pi theorem for dimensional analysis; provided a general method to derive dimensionless groups for modeling physical systems	Physics, fluid mechanics, pharmaceutical engineering	Pi theorem, dimensional analysis, similarity principles, physical modeling, scaling laws	[61]
1911	Research article	Introduced the method of zero-dimension variables for aerodynamic analysis; now foundational in dimensional analysis and widely applied in pharmaceutical process modelling and scale-up	Aerodynamics, fluid mechanics, pharmaceutical engineering, chemical process modelling	Pi theorem, dimensional analysis, scale-up, dimensionless variables, physical modelling, aerodynamics	[62]
1892	Research article	Introduces the laws of similarity and dimensional analysis, applicable to process scale-up, drug release system design, and technology transfer in the pharmaceutical industry	Pharmaceutical industry and manufacturing processes	Dimensional analysis, laws of similarity, process scale-up, technology transfer, physical-mathematical modelling	[63]
